# Real-world effectiveness of early intervention with fixed-dose tiotropium/olodaterol vs tiotropium in Japanese patients with COPD: a high-dimensional propensity score–matched cohort analysis

**DOI:** 10.1186/s12931-021-01776-y

**Published:** 2021-06-17

**Authors:** Shigeo Muro, Masaru Suzuki, Shuhei Nakamura, Jocelyn Ruoyi Wang, Elizabeth M. Garry, Wataru Sakamoto, Sabrina de Souza

**Affiliations:** 1 Department of Respiratory Medicine, Nara Medical University, 840, Shijo-cho, Kashihara-shi, Nara 634-8522 Japan; 2 Department of Respiratory Medicine, Faculty of Medicine, Hokkaido University, Sapporo, Japan; 3 Nippon Boehringer Ingelheim Co., Ltd., Tokyo, Japan; 4 Science, Aetion Inc., Boston, MA USA; 5 Boehringer Ingelheim International GmbH, Ingelheim, Germany

**Keywords:** Claims database, COPD exacerbation, COPD maintenance therapy, Japan, Real-world evidence, Tiotropium/olodaterol, Triple therapy

## Abstract

**Background:**

Escalation to triple therapy (long-acting muscarinic antagonist/β_2_-agonist, inhaled corticosteroid [ICS]) in chronic obstructive pulmonary disorder (COPD) is recommended for patients on LAMA/LABA combinations with frequent exacerbations and severe symptoms. An extended time-to-escalation to triple therapy suggests patients are in a stable condition and is an indicator of treatment effectiveness. No studies in Japanese clinical practice have compared the effectiveness of LAMA/LABA fixed-dose combination therapies with LAMA monotherapy in terms of time-to-escalation to triple therapy. The primary objective of this real-world study in Japan was to compare time-to-escalation to triple therapy among new users of tiotropium/olodaterol or tiotropium monotherapy for COPD without asthma.

**Methods:**

In this active-comparator cohort study, new users of tiotropium/olodaterol (n = 1436) and tiotropium monotherapy (n = 5352) were identified from a large Japanese hospital-based database (Medical Data Vision Co., Ltd., Tokyo; prespecified study period: 1 April 2015 to 31 March 2019); patients in each group were matched 1:1 using high-dimensional propensity scores (hdPS). The primary outcome was time-to-escalation to triple therapy.

**Results:**

For the prespecified study period in the hdPS-matched cohort, escalation to triple therapy was infrequent among new users of tiotropium/olodaterol (n = 1302, 7 escalation events) and tiotropium monotherapy (n = 1302, 8 escalation events). The difference in time-to-escalation to triple therapy between groups was not statistically significant (median [interquartile range]: 28 days [15.0–139.2] for tiotropium monotherapy vs 193 days [94.5–302.0] for tiotropium/olodaterol; hazard ratio: 0.89; 95% CI: 0.32–2.46). Similar findings (hazard ratio: 0.71; 95% Cl: 0.36–1.40) were observed in a post hoc analysis, which extended the study period by 1 year to 31 March 2020. Risks of first moderate and/or severe COPD exacerbation were lower for tiotropium/olodaterol than tiotropium monotherapy (between-group differences not significant). There were no significant between-group differences for the risks of all-cause inpatient mortality, major adverse cardiovascular events, and first use of home oxygen therapy.

**Conclusions:**

ICS monotherapy or ICS/LABA added to tiotropium or tiotropium/olodaterol is limited in Japanese clinical settings. The number of escalations to triple therapy was very limited in the dataset and there was insufficient power to detect differences between the treatment groups in the primary hdPS-matched cohort.

**Supplementary Information:**

The online version contains supplementary material available at 10.1186/s12931-021-01776-y.

## Background

Chronic obstructive pulmonary disease (COPD) is a preventable and highly prevalent respiratory disease that is associated with substantial morbidity and mortality worldwide [1]. In Japan, the prevalence of COPD among individuals aged ≥ 40 years is estimated to be 8.6% [2]. International documents or guidelines, including those from the Global Initiative for Chronic Obstructive Lung Disease (GOLD) and the Japanese Respiratory Society guidelines, recommend bronchodilation with long-acting muscarinic antagonists (LAMA) and/or long-acting β_2_-agonists (LABA) as first-line maintenance therapy for patients with moderate-to-severe COPD [3–6]. The benefits of LAMA/LABA fixed-dose combination inhalers for patients with COPD compared with LAMA or LABA monotherapy have been established in multiple clinical trials [7, 8], but limited data are available on the effectiveness of these inhalers in real-world clinical practice, including in Japan [9].

The clinical picture for COPD in Japan differs from that in other countries. Compared to COPD in Europe or in the USA, COPD in Japan occurs in elderly patients with relatively lower body mass index (BMI) and in those with radiological emphysema [10–16]. In addition, the rates of COPD exacerbation and use of inhaled corticosteroid (ICS) in Japan are lower than in other countries [15, 17]. Fixed-dose tiotropium/olodaterol (LAMA/LABA) was approved for maintenance bronchodilation in Japanese patients with COPD in September 2015. Clinical trials conducted in multiple countries, including Japan, have shown that patients treated with tiotropium/olodaterol have significantly greater improvements in lung function and health-related quality of life compared with tiotropium monotherapy [10, 18], and significantly greater improvements in physical activity and reduced sedentary time in Japanese patients [12, 19]. In the DYNAGITO trial, the subgroup of Japanese patients who received tiotropium/olodaterol had a 29% numerically lower rate of moderate-to-severe COPD exacerbations compared with tiotropium monotherapy (rate ratio 0.71; 99% CI: 0.46–1.10; p = 0.0434) [13]. Triple therapy with a LAMA/LABA combination and an ICS is typically reserved for patients who experience frequent exacerbations while taking a LAMA and/or LABA and for patients with high blood eosinophil levels [3]. However, ICSs are associated with an increased risk of adverse effects, including pneumonia [20, 21], and although the Japanese Respiratory Society guideline previously recommended the addition of ICS to a LAMA/LABA for patients with repeated COPD exacerbations [22], as of 2018, the addition of ICS is now limited to patients with COPD and asthmatic features only [4]. One real-world pharmacy claims study in the USA suggests that patients with COPD who initiate a combination LAMA/LABA escalate to triple therapy more slowly and have a lower risk of escalation than those who initiate tiotropium monotherapy [9]. To date, no studies have been conducted in Japanese clinical practice to compare the effectiveness of tiotropium/olodaterol with tiotropium monotherapy in terms of escalation to triple therapy.

The primary objective of this real-world study was to compare the time-to-escalation to triple therapy among Japanese patients with COPD without asthma who were new users of tiotropium/olodaterol or tiotropium monotherapy. A secondary objective was to assess the time to the first moderate or severe COPD exacerbation in relation to tiotropium/olodaterol or tiotropium monotherapy in a real-world setting. Patients in each treatment group were matched using high-dimensional propensity scores (hdPS), which adjust for the large number of covariates that may act as proxies for unobserved variables in healthcare databases and can therefore be an effective means of controlling for confounding [23].

## Methods

### Study design and setting

This was a retrospective, new-user, active-comparator cohort study using hdPS to match patients in the comparator groups. Patient data were sourced from a large hospital-based database (Medical Data Vision Co., Ltd. [MDV], Tokyo, Japan) [24], which includes administrative claims and Diagnosis Procedure Combination (DPC) data from hospitalizations and outpatient visits of patients attending hospitals that participate in the DPC system. DPC hospitals in Japan provide acute-phase inpatient care as well as other medical care and, as of 31 March 2019, the MDV database included 25.1 million patients and 374 DPC hospitals. The study was conducted in accordance with the Japanese Ethical Guidelines for Medical and Health Research Involving Human Subjects and a protocol that was approved by an Independent Ethics Committee (Japan Conference of Clinical Research). Patient informed consent was not required because the study used retrospective de-identified data.

**Fig. 1:**
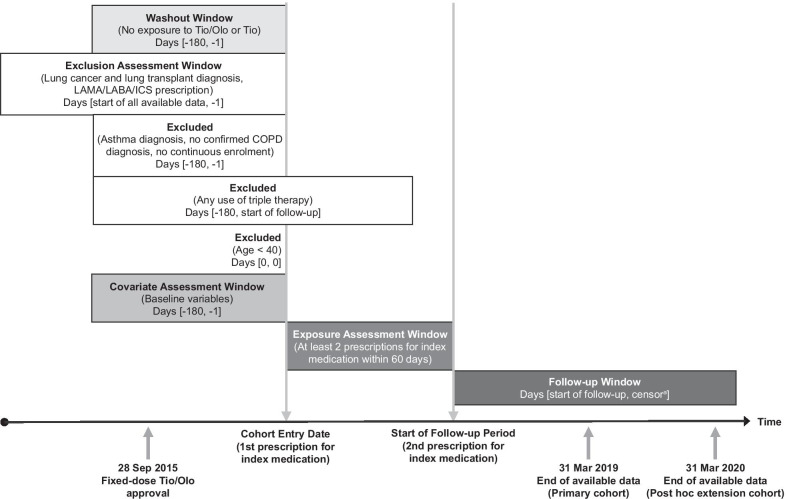
Study design. ^a^The follow-up ends at the earliest occurrence of the outcome, or at inpatient death, disenrollment, a maximum of 360 days, or the end of the study period. *COPD* chronic obstructive pulmonary disorder, *ICS* inhaled corticosteroid, *LABA* long-acting β_2_-agonist, *LAMA* long-acting muscarinic antagonist, *Tio* tiotropium, *Tio/Olo* tiotropium/olodaterol

The prespecified study period ranged from 1 April 2015 to 31 March 2019 (Fig. 1) and included the cohort entry date (index date; first prescription for fixed-dose tiotropium/olodaterol [Spiolto® Respimat®, Boehringer Ingelheim International GmbH, Ingelheim, Germany] or tiotropium monotherapy administered as an inhalation Soft mist inhaler or powder [Spiriva® Respimat® or Spiriva® Handihaler®, Boehringer Ingelheim International GmbH, Ingelheim, Germany]), a 180-day baseline period (ie, 180 days before the cohort entry date and at least 180 days after approval of fixed-dose tiotropium/olodaterol), and a follow-up period, starting at the second prescription for tiotropium/olodaterol or tiotropium within 60 days of the first prescription and ending at the earliest use of any triple therapy, patient death, discontinuation, end of the study period, or a maximum follow-up of 1 year, whichever occurred first. Triple therapy was defined as any fixed dose or concurrent use for 30 consecutive days of LAMA/LABA/ICS, including any LAMA/LABA fixed-dose combination plus any single ICS formulation; any single LAMA formulation plus any ICS/LABA fixed-dose combination; and any single LAMA formulation plus any single LABA formulation plus any single ICS formulation, regardless of the reason for prescription of the ICS.

During analysis of the primary dataset, data for 1 additional year (from 1 April 2015 to 31 March 2020) became available. Therefore, a post hoc analysis of these additional data was conducted (post hoc extension period).

### Study cohorts

The primary analysis cohort included all patients in the MDV database with a confirmed diagnosis of COPD (International Statistical Classification of Diseases and Related Health Problems, 10th Revision [ICD-10] codes J41, J43, J44) who received a first prescription for tiotropium/olodaterol or tiotropium monotherapy from the start until 90 days before the end of the respective study periods. In addition, patients were required to have a second claim for index medication within 60 days of cohort entry to ensure primary adherence to the index medication. Patients aged < 40 years at the time of cohort entry were excluded during patient selection to minimize the likelihood of including those with a misdiagnosis (eg, asthma or bronchitis coded as COPD). In addition, all patients with a confirmed diagnosis of asthma (ICD-10: J45) during the baseline period or a confirmed diagnosis of lung cancer (ICD-10: C34, D02.2, Z80.1, Z85.1) or lung transplant (Health claim code: 150317670, 150322510, 150322610, 150336510, 150336610, 150336710, 150399270) at any time before cohort entry were excluded during patient selection. Other exclusion criteria were: a prescription for any LAMA, LABA, or ICS maintenance therapy (alone or in combination) during the baseline period before cohort entry for maintenance treatment with duration > 30 days or any prescription within 30 days before cohort entry; < 180 days of continuous enrolment during the baseline period; initiation of both index medications at the same time on cohort entry; and use of triple therapy during the baseline period or between the cohort entry date and the day before the start of follow-up.

### Variables

#### Baseline covariates

The hdPS for the probability of initiating tiotropium/olodaterol compared with tiotropium monotherapy was computed using logistic regression [23] and included predefined demographic variables of age, sex, index calendar year, hospital size (< 199 beds, 200–499 beds, ≥ 500 beds), concomitant medications and comorbidities, number of COPD exacerbations, all-cause hospitalization, hospitalization due to respiratory conditions, and covariates identified by the hdPS multi-step algorithm from relevant data fields related to disease (ICD-10 codes) [23], inpatient and outpatient health claim codes, and MDV laboratory codes during the baseline period. The final hdPS was then used to match one new user of tiotropium/olodaterol to one new user of tiotropium monotherapy with the same propensity score caliper of 1% [25].

#### Exposure

Exposure groups were identified according to the brand name of the first two prescriptions of each drug within 60 days. Each prescription was assumed to be sufficient for 14 days for Spiolto Respimat 28 puffs, and for 30 days for Spiriva Handihaler 18 µg, Spiriva 2.5 µg/Respimat 60 puffs, and Spiolto Respimat 28 puffs and 60 puffs. A gap of 14 days between the end of supply of the first prescription and the start of a subsequent prescription was allowed.

#### Study outcomes

The primary outcome was the time-to-escalation to any LAMA/LABA/ICS triple therapy (fixed-dose or concurrent use) during the follow-up period. Secondary outcomes were the time to the first moderate and/or severe COPD exacerbation during the follow-up period. Moderate exacerbations were defined as an outpatient visit with a confirmed COPD diagnosis in any field and a prescription for an oral corticosteroid or antibiotic for respiratory infection. Severe exacerbations were defined as hospitalization with a primary diagnosis of COPD [26]. Other outcomes included time-to-all-cause inpatient mortality, major adverse cardiovascular events (MACE), and use of home oxygen therapy during the follow-up period. A MACE included ischaemic stroke (ICD-10: I60.x, I61.x, I63.x, I67.8) [27–29], myocardial infarction (ICD-10: I21.x, I22) [30, 31], or inpatient death (ICD-10: O96.x, O97.x, I46.1, R96, R98, R99). Home oxygen therapy was defined according to various health claim codes related to use of home/domiciliary oxygen therapy. In this study, ICD-10 refers to the World Health Organization classification system and not the ICD-10 clinical modification (ICD-10-CM) system.

### Statistical analysis

The sample size for this study was based on a retrospective analysis of an insurance claims database of patients with COPD in the USA [9]. The percentage of patients who escalated to triple therapy within 1 year of starting treatment with a LAMA (tiotropium monotherapy) or LAMA/LABA (umeclidinium/vilanterol) was 10% and 5%, respectively. Using these results, we estimated that 1856 patients (141 escalation events) were required to detect a difference between groups in the time-to-escalation to triple therapy (hazard ratio [HR]: 1.73) with 90% power and a two-sided alpha of 0.05 in the current study.

Descriptive statistics are reported as n (%), mean (standard deviation), and median (interquartile range [IQR]) as applicable. Except for baseline characteristics, all variables with > 75% missing data were excluded from the analyses.

A Cox proportional hazard regression model with an intention-to-treat censoring approach was used to compare time-to-event outcomes during the follow-up period in each treatment group. Time-to-event outcomes are reported as HRs with 95% confidence intervals (95% CI). In addition, time-to-escalation to triple therapy was assessed using Kaplan–Meier curves. The rate of escalation to triple therapy in each treatment group was defined as the number of patients who escalated to triple therapy (ie, an escalation event) divided by the total number of patient-years at risk during follow-up.

Sensitivity analyses were conducted as follows. First, to decrease the stringency of the eligibility criteria and potentially increase the sample size, only patients who had one prescription for an index medication (rather than two prescriptions within 60 days) were included in sensitivity cohort 1. Second, the as-treated approach was used. In addition to the censoring rule implemented in the intention-to-treat approach, sensitivity cohort 2 included patients who, in addition to outcome, death, or end of available data, were censored after they discontinued index medication or switched from one index medication to the other. Third, to increase specificity, sensitivity cohort 3 excluded patients with the ICD-10 J41 code for chronic bronchitis as this code is commonly used when prescribing a mucolytic to patients who do not have chronic bronchitis. Fourth, sensitivity cohort 4 included patients with COPD and a diagnosis code for asthma with doubt (ie, no prescriptions for asthma-specific treatment such as ICS or ICS/LABA). Fifth, to minimize the effect of patients with overlapping symptoms of asthma and COPD, sensitivity cohort 5 included censored patients on the date of an asthma diagnosis during the follow-up period.

All analyses were conducted using the Aetion Evidence Platform® (2020) version R4.2. (Aetion Inc., New York, NY, USA), a software platform for real-world data analysis, which has been validated for a range of studies [32]. The platform was used to select the cohorts, create the analytic variables, and calculate the hdPS.

## Results

### Patient selection and baseline characteristics

**Fig. 2:**
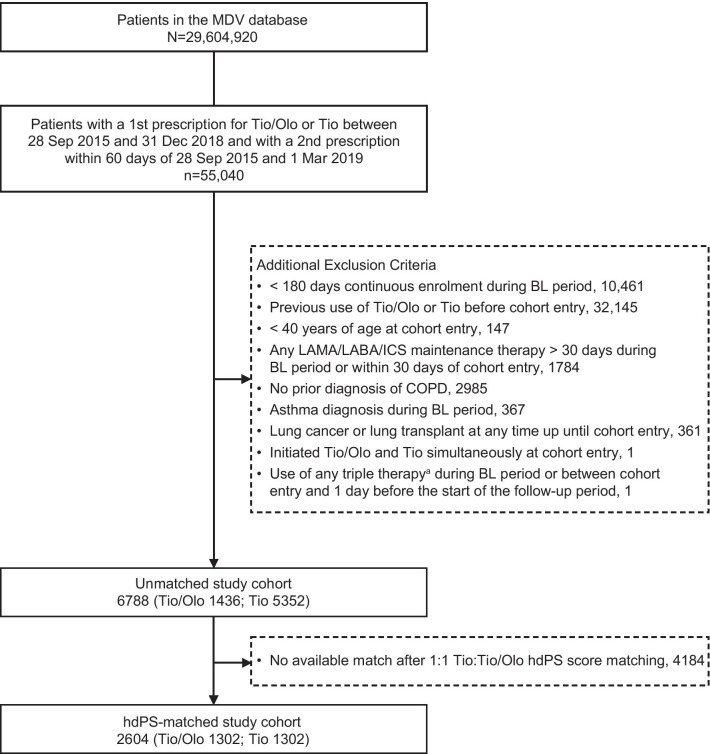
Patient selection for the prespecified study period (1 April 2015 to 31 March 2019). ^a^Triple therapy included any fixed-dose or concurrent use of LAMA, LABA, and ICS. *BL* baseline, *COPD* chronic obstructive pulmonary disorder, *hdPS* high-dimensional propensity score, *ICS* inhaled corticosteroid, *LABA* long-acting β_2_-agonist, *LAMA* long-acting muscarinic antagonist, *MDV* Medical Data Vision Co., Ltd. database, *Tio* tiotropium, *Tio/Olo* tiotropium/olodaterol

For the prespecified study period, of the 55,040 patients in the MDV database who were eligible for inclusion in the primary analysis, 1436 new users of tiotropium/olodaterol and 5352 new users of tiotropium monotherapy met all eligibility criteria and were included in the unmatched study cohort (Fig. 2). After computation of hdPS, 1302 new users of tiotropium/olodaterol were matched 1:1 to 1302 new users of tiotropium monotherapy. For the post hoc extension study period, there were 1860 new users of tiotropium/olodaterol and 6505 new users of tiotropium monotherapy who met all eligibility criteria and were included in the unmatched study cohort (Additional file 1: Table S1a). After computation of hdPS, 1723 new users of tiotropium/olodaterol were matched 1:1 to 1723 new users of tiotropium monotherapy. The median (IQR) follow-up time for the hdPS-matched cohorts among new users of tiotropium/olodaterol and tiotropium monotherapy were 327 (138–360) and 338 (154–360) days, respectively, in the prespecified study period and were 360 (139–360) and 360 (138–360) days, respectively, in the post hoc extension period.

Patient demographics and characteristics of the primary analysis cohort were generally well balanced before matching, with most patients in both study periods entering the study from 2017 onwards (Table 1). However, in the unmatched cohort, new tiotropium/olodaterol users had indicators of greater COPD severity compared with new tiotropium users (ie, more patients in the tiotropium/olodaterol group had COPD prescriptions for oral or injected corticosteroids and for cough and cold treatments, and more COPD exacerbations, all-cause hospitalizations, and hospitalizations due to respiratory conditions). Across all cohorts, the incidence of pneumonia at baseline was approximately 20% (Table 1).

**Table 1:** Baseline characteristics in the prespecified study period and post hoc extension study period

Variable	Prespecified study period (1 April 2015 to 31 March 2019)				Post hoc extension period (1 April 2015 to 31 March 2020)			
	hdPS-matched cohort		Unmatched cohort		hdPS-matched cohort		Unmatched cohort	
	Tio n = 1302	Tio/Olo n = 1302	Tio n = 5352	Tio/Olo n = 1436	Tio n = 1723	Tio/Olo n = 1723	Tio n = 6505	Tio/Olo n = 1860
Age at cohort entry, mean (SD), years	75.1 (8.9)	75.2 (8.5)	75.6 (8.9)	75.2 (8.4)	75.6 (8.8)	75.4 (8.5)	75.6 (8.9)	75.4 (8.5)
Male, n (%)	1140 (87.6)	1148 (88.2)	4573 (85.4)	1273 (88.6)	1524 (88.5)	1520 (88.2)	5543 (85.2)	1649 (88.7)
Year of cohort entry, n (%)								
2015 + 2016	86 (6.6)	97 (7.5)	2196 (41.0)	99 (6.9)	86 (5.0)	84 (4.9)	2227 (34.2)	84 (4.5)
2017	586 (45.0)	581 (44.6)	1815 (33.9)	617 (43.0)	527 (30.6)	528 (30.6)	1860 (28.6)	546 (29.4)
2018	594 (45.6)	590 (45.3)	1282 (24.0)	679 (47.3)	566 (32.8)	569 (33.0)	1415 (21.8)	605 (32.5)
2019	36 (2.8)	34 (2.6)	59 (1.1)	41 (2.9)	519 (30.1)	517 (30.0)	955 (14.7)	595 (32.0)
Hospital size by bed number, n (%)								
< 199 beds	144 (11.1)	129 (9.9)	706 (13.2)	138 (9.6)	175 (10.2)	162 (9.4)	885 (13.6)	171 (9.2)
200–499 beds	685 (52.6)	709 (54.5)	3031 (56.6)	760 (52.9)	924 (53.6)	929 (53.9)	3640 (56.0)	980 (52.7)
≥ 500 beds	472 (36.3)	462 (35.5)	1575 (29.4)	536 (37.3)	622 (36.1)	630 (36.6)	1937 (29.8)	707 (38.0)
Blood eosinophil count %, mean (SD)^a^	2.69 (2.56)^b^	2.89 (2.71)^b^	3.07 (3.13)^c^	2.93 (2.84)^c^	2.94 (3.28)^d^	2.99 (2.83)^d^	3.05 (3.28)^e^	2.93 (2.79)^e^
Respiratory events and medications, n (%)								
All-cause hospitalization	524 (40.2)	535 (41.1)	2013 (37.6)	617 (43.0)	742 (43.1)	745 (43.2)	2507 (38.5)	846 (45.5)
Hospitalization due to respiratory condition	475 (36.5)	485 (37.3)	1798 (33.6)	560 (39.0)	660 (38.3)	670 (38.9)	2240 (34.4)	762 (41.0)
Cough and cold preparations	366 (28.1)	364 (28.0)	1240 (23.2)	436 (30.4)	485 (28.1)	477 (27.7)	1489 (22.9)	552 (29.7)
Oral/injected corticosteroids	220 (16.9)	219 (16.8)	711 (13.3)	263 (18.3)	289 (16.8)	291 (16.9)	860 (13.2)	337 (18.1)
COPD exacerbations	181 (13.9)	166 (12.7)	530 (9.9)	192 (13.4)	221 (12.8)	220 (12.8)	643 (9.9)	256 (13.8)
COPD exacerbations + oral/injected corticosteroids	131 (10.1)	130 (10.0)	384 (7.2)	148 (10.3)	163 (9.5)	183 (10.6)	466 (7.2)	205 (11.0)
Other concomitant therapy, n (%)								
Antihypertensives/diuretics	443 (34.0)	457 (35.1)	1738 (32.5)	520 (36.2)	635 (36.9)	629 (36.5)	2175 (33.4)	708 (38.1)
Antithrombotic agents (including aspirin/DOAC)	382 (29.3)	391 (30.0)	1523 (28.5)	460 (32.0)	528 (30.6)	531 (30.8)	1909 (29.3)	601 (32.3)
Antiepileptics/psycholeptics/psychoanaleptics (hypnotics/sedatives)^f^	325 (25.0)	315 (24.2)	1188 (22.2)	371 (25.8)	439 (25.5)	431 (25.0)	1475 (22.7)	497 (26.7)
Hypnotics/sedatives (N5B)^g^	207 (15.9)	206 (15.8)	768 (14.3)	244 (17.0)	292 (17.0)	294 (17.1)	960 (14.8)	330 (17.7)
Lipid-lowering agents	182 (14.0)	189 (14.5)	698 (13.0)	208 (14.5)	268 (15.6)	259 (15.0)	893 (13.7)	290 (15.6)
Antirheumatics, non-steroidal	172 (13.2)	171 (13.1)	653 (12.2)	215 (15.0)	240 (13.9)	227 (13.2)	771 (11.9)	269 (14.5)
Comorbidities before cohort entry, n (%)								
Hypertension	237 (18.2)	253 (19.4)	1069 (20.0)	288 (20.1)	339 (19.7)	354 (20.5)	1332 (20.5)	394 (21.2)
Pneumonia	246 (18.9)	244 (18.7)	911 (17.0)	286 (19.9)	339 (19.7)	344 (20.0)	1119 (17.2)	399 (21.5)
Heart failure	200 (15.4)	203 (15.6)	821 (15.3)	229 (15.9)	265 (15.4)	274 (15.9)	1017 (15.6)	297 (16.0)
Any cancer (except non-melanoma skin cancer)	147 (11.3)	145 (11.1)	469 (8.8)	178 (12.4)	208 (12.1)	204 (11.8)	580 (8.9)	236 (12.7)
Gastroesophageal reflux disease	141 (10.8)	142 (10.9)	672 (12.6)	165 (11.5)	198 (11.5)	211 (12.2)	796 (12.2)	235 (12.6)
Type 2 diabetes mellitus	131 (10.1)	126 (9.7)	481 (9.0)	151 (10.5)	184 (10.7)	188 (10.9)	594 (9.1)	215 (11.6)
Chronic bronchitis	65 (5.0)	74 (5.7)	264 (4.9)	86 (6.0)	79 (4.6)	89 (5.2)	314 (4.8)	100 (5.4)

*COPD* chronic obstructive pulmonary disease, *DOAC* direct oral anticoagulant, *hdPS* high-dimensional propensity score, *n* number of patients, *SD* standard deviation, *Tio* tiotropium, *Tio/Olo* tiotropium/olodaterol

^a^Blood eosinophil count was not included in the hdPS models

^b^Data only included for 50 (3.8%) patients in the tiotropium group and 68 (5.2%) patients in the tiotropium/olodaterol group

^c^Data only included for 178 (3.3%) patients in the tiotropium group and 76 (5.3%) patients in the tiotropium/olodaterol group

^d^Data only included for 63 (3.7%) patients in the tiotropium group and 81 (4.7%) patients in the tiotropium/olodaterol group

^e^Data only included for 211 (3.2%) patients in the tiotropium group and 85 (4.6%) patients in the tiotropium/olodaterol group

^f^Includes hypnotics/sedatives

^g^Not included in the hdPS models

For the prespecified study period, in the hdPS-matched cohort, patient demographics and characteristics were well balanced and the majority of patients were elderly (mean age 75 years) men with a low rate of COPD exacerbations. During the baseline period, 13.3% of patients had at least one COPD exacerbation (tiotropium/olodaterol: 166 events; tiotropium monotherapy: 181 events), over one-third (approximately 37%) of patients had been hospitalized for a respiratory condition, 28% were prescribed cough and cold preparations, approximately 17% were prescribed oral or injected corticosteroids, and 10% had at least one COPD exacerbation and were also prescribed oral or injected corticosteroids. Approximately 30% of patients were prescribed antithrombotic agents, including temporary use of aspirin and direct oral anticoagulants. Approximately 25% of patients were prescribed antiepileptics, psycholeptics, or psychoanaleptics, and 15.9% in the tiotropium group and 15.8% in the tiotropium/olodaterol group were prescribed hypnotics/sedatives. In general, these baseline characteristics were similar for patients in the post hoc extension cohort (Table 1).

For the prespecified and post hoc extension sensitivity analysis cohorts (Additional file 1: Table S1a,b), sensitivity analysis 1 (first prescription only) included a greater number of patients than the primary cohort, as intended; sensitivity analyses 2 (as-treated) and 5 (excluding patients with overlapping symptoms of asthma and COPD) included the same number of patients as the primary cohort; and sensitivity analyses 3 (COPD codes J34 and J44, bronchitis J41 excluded) and 4 (COPD and asthma with doubt) included similar numbers of patients as in the primary cohort.

### Escalation to triple therapy

The rate of escalation to triple therapy was very low in both treatment groups in the hdPS-matched and unmatched cohorts during the prespecified study period, with less than 1% of patients escalating to triple therapy in the primary hdPS-matched cohort (Table 2). There were 15 escalation events: seven in the tiotropium/olodaterol (0.54%) group and eight in the tiotropium group (0.61%). The number of escalations to triple therapy in Japanese patients in this study was less than expected and yielded insufficient power to detect significant differences between treatment groups.

**Table 2:** Escalation to triple therapy (fixed-dose or any concurrent ICS/LAMA/LABA) during each study period

Variable	Prespecified study period (1 April 2015 to 31 March 2019)		Post hoc extension study period (1 April 2015 to 31 March 2020)	
	Tio	Tio/Olo	Tio	Tio/Olo
hdPS-matched cohort (primary analysis)				
Number of patients	1302	1302	1723	1723
Number of events	8	7	20	14
Number of patient-years	919	899	1211	1203
Events per 1000 patient-years (95% CI)	8.71 (4.11–16.43)	7.79 (3.47–15.29)	16.52 (9.28–23.75)	11.64 (5.54–17.73)
Median (IQR) time-to-escalation to triple therapy, days	28.0 (15.0–139.2)	193 (94.5–302.0)	108 (60.5–256.8)	225 (82.2–312.0)
HR for time-to-escalation (95% CI)	0.89 (0.32–2.46)		0.71 (0.36–1.40)	
Unmatched cohort				
Number of patients	5352	1436	6505	1860
Number of escalation events	30	8	49	20
Number of patient-years	4180	981	5065	1291
Events per 1000 patient-years (95% CI)	7.18 (4.61–9.75)	8.16 (3.52–16.07)	9.67 (6.97–12.38)	15.49 (8.70–22.28)
Median (IQR) time-to-escalation to triple therapy, days	100.5 (28.0–196.8)	191 (117.2–292.0)	112 (56.0–245.0)	255.5 (176.8–311.5)
HR for time-to-escalation (95% CI)	1.11 (0.51–2.43)		1.60 (0.95–2.69)	

*CI* confidence interval, *hdPS* high-dimensional propensity score, *HR* hazard ratio, *ICS* inhaled corticosteroid, *IQR* interquartile range, *LABA* long-acting β_2_-agonist, *LAMA* long-acting muscarinic antagonist, *Tio* tiotropium, *Tio/Olo* tiotropium/olodaterol

**Fig. 3:**
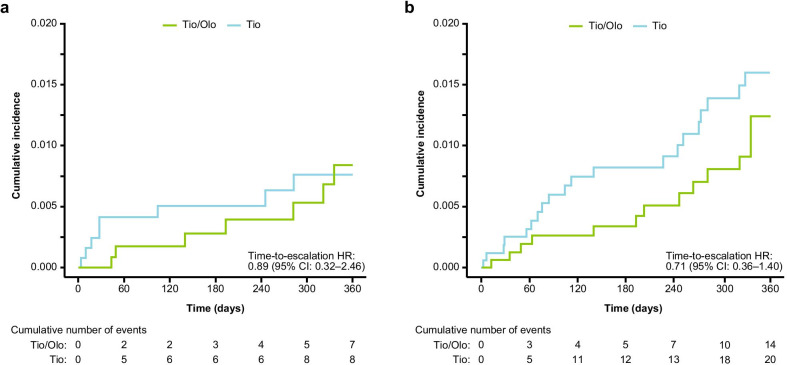
Kaplan–Meier curves of escalation to triple therapy (fixed-dose or concurrent LAMA/LABA/ICS) for the hdPS-matched cohort. Data are shown for the prespecified study period (1 April 2015 to 31 March 2019) (**a**) and the post hoc extension period (1 April 2015 to 31 March 2020) (**b**). *CI* confidence interval, *hdPS* high-dimensional propensity score, *HR* hazard ratio, *ICS* inhaled corticosteroid, *LABA* long-acting β_2_-agonist, *LAMA* long-acting muscarinic antagonist, *Tio* tiotropium, *Tio/Olo* tiotropium/olodaterol

For the hdPS-matched cohort, the median (IQR) time-to-escalation to triple therapy in patients was numerically shorter in the tiotropium group than in the tiotropium/olodaterol group (28 days [15.0–139.2] vs 193 days [94.5–302.0]), but the difference between treatment groups was not statistically significant (HR: 0.89; 95% CI: 0.32–2.46) (Table 2, Fig. 3a). Similarly, there were no significant differences in the time-to-escalation to triple therapy between treatment groups among the sensitivity analysis cohorts (Additional file 1: Table S2).

Although the number of escalation events more than doubled during the post hoc extension period compared with the prespecified period, the rate of escalation to triple therapy was still low in both treatment groups (Table 2). In the hdPS-matched cohort, there were 34 escalation events: 14 in the tiotropium/olodaterol group (0.81%) and 20 in the tiotropium group (1.16%). In the hdPS-matched cohort, the median (IQR) time-to-escalation to triple therapy was numerically shorter in the tiotropium group than in the tiotropium/olodaterol group (108 days [60.5–256.8] vs 225 days [82.2–312]) and, similar to the prespecified cohort, there were no statistically significant differences in the time-to-escalation to triple therapy between treatment groups in the hdPS-matched cohorts (HR: 0.71; 95% CI: 0.36–1.40) (Table 2, Fig. 3b).

### COPD exacerbations and other secondary outcomes

**Fig. 4:**
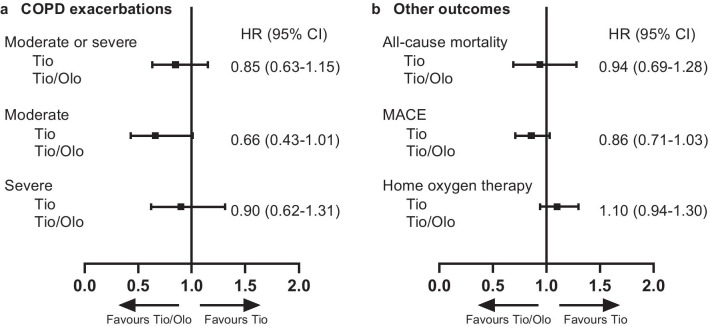
Risk of a first COPD exacerbation (**a**) and other secondary outcomes (**b**). Data are shown for the hdPS-matched cohort (primary analysis) during the prespecified study period (1 April 2015 to 31 March 2019). Risk was assessed on the time-to-event outcome. *CI* confidence interval, *COPD* chronic obstructive pulmonary disorder, *hdPS* high-dimensional propensity score, *HR* hazard ratio, *MACE* major adverse cardiovascular event, *Tio* tiotropium, *Tio/Olo* tiotropium/olodaterol

During the prespecified study period in the hdPS-matched cohort, the risks of first moderate and/or severe COPD exacerbations were lower in the tiotropium/olodaterol group than in the tiotropium monotherapy group, but the differences between groups were not statistically significant (Fig. 4a). Similar trends were observed in the sensitivity analyses (Additional file 1: Fig. S1).

During the prespecified study period in the hdPS-matched cohort, the risks of all-cause inpatient mortality, MACE, and first use of home oxygen therapy were not significantly different between the tiotropium/olodaterol and tiotropium groups (Fig. 4b). Similar trends were observed in the sensitivity analyses (Additional file 1: Fig. S2).

## Discussion

Although asthma and COPD share similar symptoms associated with reduced lung function, they require different treatment approaches [33, 34]. For asthma, ICS is the first option for treatment and the mainstay of patient management [33], whereas for COPD, ICS is carefully introduced and its use is considered a trade-off between infection risk and exacerbation control [34]. This is the first real-world study in clinical practice in Japan to assess escalation to triple therapy, which included ICS, among new users of tiotropium/olodaterol and tiotropium monotherapy in patients with COPD without asthma. The number of patients who escalated to triple therapy over the prespecified and post hoc extension periods (15 and 34, respectively) in this study was far lower than was expected [9], which resulted in insufficient power to detect differences in the time-to-escalation to triple therapy between the groups. Apart from the low number of escalation events, this study provides real-world evidence on the management of COPD and on the rate of COPD exacerbation in Japanese patients with COPD.

Although this study was focused on patients with COPD without asthma, escalation to triple therapy was counted, irrespective of whether it was prescribed for patients with COPD or newly diagnosed asthma or asthmatic features. The key reason that most likely contributed to the limited use of triple therapy in this study is that Japanese clinicians may be hesitant to prescribe ICS to patients with COPD, who are at increased risk of pneumonia. In Japan, the incidence of community-acquired pneumonia is highest in older men and is estimated to affect approximately 4–15% of men aged ≥ 65 years annually [35], and in this study occurred in approximately 20% of patients at baseline. Typical of the Japanese population with COPD [12–14, 36, 37], the patients in this study were predominantly men in their 70s with a history of infrequent COPD exacerbation and a low rate of chronic bronchitis. This is consistent with several clinical trials and observational studies that have shown that, in contrast to their European or US counterparts, Japanese patients with COPD are older, have a lower BMI, are more typically ex-smokers with longer smoking histories [10–15], and are mostly diagnosed with emphysema [16]. As older age (≥ 75 years), emphysema, low BMI, and use of ICS are all independent risk factors for pneumonia in Japan [38–40], Japanese clinicians may be hesitant to prescribe ICS to patients with COPD in real-world settings. A higher risk of pneumonia in Japanese patients with COPD is also supported by data from the Japanese population of clinical trials, where, for example, the incidence of pneumonia has been reported to be around three times higher in Japanese patients with moderate-to-severe COPD compared with non-Japanese patients [13]. Therefore, consistent with the findings from the MDV database, this higher incidence of pneumonia in the Japanese patients is likely because of the higher average age compared with Caucasians and the treatment setting in Japan, where pneumonia can be diagnosed by general practitioners who have access to radiographic imaging.

Since 2018, the Japanese COPD guidelines have limited the use of ICS to patients with COPD and asthmatic features [4, 42]. These guidelines differ from the global COPD guidelines, which no longer refer to asthma-COPD overlap [41] and, since 2020, do not limit the use of ICS to patients with concomitant asthmatic symptoms [34]. This difference in treatment guidelines may have been responsible for the smaller number of Japanese patients who escalated to triple therapy during the study period compared with studies outside of Japan where use of ICS is not limited. In addition, although this study included any fixed-dose or other combination of ICS/LAMA/LABA as triple therapy, fixed-dose ICS/LAMA/LABA only became available for use in Japan in 2019. Further, whereas the COPD exacerbation rate in this study (0.09–0.11 person-years) was consistent with the rates observed in other real-world studies in Japan [15, 36], the COPD exacerbation rate in Japan appears to be lower than in other countries [15]. Possible reasons for a lower rate of COPD exacerbation in Japan are a tendency among patients to underreport their exacerbations and because Japanese physicians may be more likely to diagnose patients with COPD and asthma-like features as having asthma [15]. Moreover, some patients who were more likely to have benefited from the addition of an ICS may have been excluded from the analyses. Current international guidelines and documents recommend exacerbation history and blood eosinophil count, together with clinical assessment, to identify patients with COPD who are likely to benefit from the addition of ICS to maintenance bronchodilation with LAMA and/or LABA [34, 43]. The proposed cut-offs for patients who may benefit from the addition of an ICS are high blood eosinophil counts of ≥ 300 cells/μL and ≥ 2 exacerbations per year [44, 45]. Among the < 10% of the hdPS-matched cohort with data available for blood eosinophil counts, mean blood eosinophil counts were low (2.69–2.89%), which suggests that patients such as those with asthma-like features may have been excluded. In addition, because the exhaled nitric oxide test, which is used to assess lung inflammation and steroid effectiveness, is covered by insurance for patients under the DPC code name for bronchial asthma in Japan, it is possible that patients with asthma-like features who underwent this test were excluded from the dataset.

In the DYNAGITO trial, where patients were selected for inclusion based on their COPD exacerbation history, the difference between tiotropium/olodaterol and tiotropium for the prevention of COPD exacerbations was not statistically significant [11]. No statistically significant differences in the risk of a first moderate-to-severe COPD exacerbation were found between treatment groups in this real-world study in Japan, but because the sample size for this study was not based on exacerbation rates, the number of COPD exacerbations observed was unlikely to provide sufficient power to detect differences between the groups. However, it was notable that the magnitude of the effect of tiotropium/olodaterol compared with tiotropium monotherapy for the risk of first moderate-to-severe COPD exacerbation in this study was consistent with that observed in the DYNAGITO Japanese subpopulation (current study: HR: 0.85; 95% CI: 0.63–1.15; DYNAGITO Japanese subpopulation: HR: 0.81; 99% CI: 0.57–1.17).

In this study, the rate of all-cause hospitalization (approximately 40% of patients) and hospitalization due to respiratory causes (37% of patients) before initiating maintenance bronchodilation in the hdPS-matched cohort was high, which suggests that patients with COPD in the MDV database may have had more severe pulmonary disease than those in outpatient settings and those who are seen by individual practitioners at small clinics. Before matching, there was an imbalance between the treatment groups for indicators of more severe pulmonary lung function and, although the imbalance was minimized after matching, very small differences between groups in all-cause hospitalization and hospitalization due to respiratory causes remained. Hence, it is conceivable that, even though patients were hdPS matched, those in the tiotropium/olodaterol treatment group may have had slightly greater COPD severity than those in the tiotropium monotherapy group. Because very few exacerbations were observed in this cohort, it is possible that even small differences in disease severity between treatment groups may have affected the direction and magnitude of treatment outcomes [46].

The safety of tiotropium/olodaterol compared with tiotropium monotherapy has been assessed in a pooled analysis of data from three large, 52-week, randomized controlled trials of patients with moderate-to-severe COPD [47]. In the pooled analysis of 9942 patients with COPD, there were no significant differences in the incidence of MACE between tiotropium/olodaterol and tiotropium (2.11 vs 2.22 per 100 patient-years), and the rate of all-cause mortality was low (2.26 vs 2.44 per 100 patient-years, respectively). Although several clinical trials have assessed the safety of tiotropium/olodaterol in Japanese patients, the sample sizes have been relatively small [13, 18, 48]. This real-world study, therefore, is the largest to provide an assessment of safety outcomes associated with the use of tiotropium/olodaterol in Japanese patients.

Studies conducted in real-world clinical practice are needed to provide clinically relevant information on real-world treatment practices and patient responses. In this study, use of an administrative claims database allowed the analysis of a large number of patients and, by using hdPS matching, we could adjust for potential known and unknown confounders of treatment effects while maintaining a balance in baseline demographics and clinical characteristics between the treatment groups. Although patients were matched against more than 200 variables, the MDV database has limited information on patient characteristics such as BMI, laboratory tests, and vaccination history, and does not collect data on lung function and symptoms or treatment outcomes [49]. Therefore, the potential for unmeasured or residual confounding could not be eliminated. The findings from this study can be widely generalized to Japanese patients with COPD because the MDV dataset includes patients from a wide geographic area across Japan. In addition, the Japanese medical system allows patients to visit hospitals for primary care, so patients included in the MDV dataset are not limited to acute care only.

There are several limitations that should be considered when interpreting the findings from this study. First, the overestimation of the underlying rate of escalation to triple therapy among Japanese patients in this study population resulted in insufficient power to detect differences between the treatment groups. Although the sample size calculation was based on published data that were currently available, there were unexpected differences in characteristics between the population set for the calculation and Japanese patients in this real-world setting. Second, although MDV is one of the largest hospital-based databases in Japan, including approximately 25 million patients (ie, 25% of the Japanese population), it is not population based and does not include all hospitals in Japan. Therefore, the MDV dataset is not representative of the minority of Japanese patients with COPD who visit small clinics. Third, as data are collected under real-world conditions, measurements and/or investigations are not standardized and patients’ comorbidities and clinical characteristics may be misclassified. Despite this, variance in the quality of data between hospitals is likely to be random. Fourth, the MDV database does not provide information on individual treatment regimens, which means that a prescription claim in the MDV database may not be representative of actual treatment. We attempted to mitigate this concern by requiring patients to have two prescriptions for index medication within 60 days. Although this approach may have contributed to selection bias, analyses of the sensitivity cohort that only included patients with one prescription for an index medication suggested that the effects of potential selection bias were likely to be minimal. Fifth, because healthcare is nationalized in Japan, the notion of enrolment is not applicable, and all patients were assumed to be observable throughout the study period. To address this limitation, we used a proxy measure for enrolment, which was defined as the first patient encounter, and for the end of enrolment, defined as the last patient encounter. As patient data would still be included even if treatment discontinuations or deaths occurred outside the MDV hospital network, this limitation could also possibly lead to an underestimation of discontinuations or deaths. Nonetheless, because the median follow-up time was not different between treatment groups in the prespecified and post hoc extension periods, the effects of unrecorded treatment discontinuation or death are unlikely to be clinically relevant. Finally, patients in the MDV database are not tracked between hospitals; therefore, patients who were prescribed treatment at more than one hospital may have been counted more than once.

## Conclusions

In conclusion, the findings from this real-world study suggested that, compared with new users of tiotropium, new users of tiotropium/olodaterol had a numerically lower probability of escalating to triple therapy or experiencing moderate or severe COPD exacerbations. However, as the numbers of escalations were overestimated in this Japanese clinical setting, there was insufficient power to detect differences in time-to-escalation between the treatment groups in the primary hdPS-matched cohort. In addition, no significant differences in the risk of inpatient mortality, MACE, or use of home oxygen therapy among new users of tiotropium/olodaterol compared with new users of tiotropium could be detected.

## Supplementary Information

**Additional file 1: Table S1a.** Patient selection in the primary and sensitivity analysis cohorts. **Table S1b.** Patient selection in the sensitivity analysis cohorts for the post hoc extension study period (from 28 September 2015 to 31 March 2020). **Table S2**. Sensitivity analyses for escalation to triple therapy (fixed-dose or concurrent ICS/LAMA/LABA) during the prespecified study period (from 28 September 2015 to 31 March 2019). **Fig. S1.** Sensitivity analyses for the time-to-first COPD exacerbation in the hdPS-matched cohort during the prespecified study period (1 April 2015 to 31 March 2019). CI, confidence interval; COPD, chronic obstructive pulmonary disorder; hdPS, high-dimensional propensity score; HR, hazard ratio; SA, sensitivity analysis; Tio, tiotropium; Tio/Olo, tiotropium/olodaterol. **Fig. S2.** Sensitivity analyses for the time-to-event for all-cause inpatient mortality, first MACE, and first use of home oxygen therapy in the hdPS-matched cohort during the prespecified study period (1 April 2015 to 31 March 2019). CI, confidence interval; hdPS, high-dimensional propensity score; HR, hazard ratio; MACE, major adverse cardiovascular event; SA, sensitivity analysis; Tio, tiotropium; Tio/Olo, tiotropium/olodaterol.

## Data Availability

To ensure independent interpretation of clinical study results, Boehringer Ingelheim grants all external authors access to all relevant material, including participant-level clinical study data, and relevant material as needed by them to fulfill their role and obligations as authors under the ICMJE criteria. Furthermore, clinical study documents (e.g. study report, study protocol, statistical analysis plan) and participant clinical study data are available to be shared after publication of the primary manuscript in a peer-reviewed journal and if regulatory activities are complete and other criteria met per the BI Policy on Transparency and Publication of Clinical Study Data: https://trials.boehringer-ingelheim.com/. Prior to providing access, documents will be examined, and, if necessary, redacted and the data will be de-identified, to protect the personal data of study participants and personnel, and to respect the boundaries of the informed consent of the study participants. Clinical Study Reports and Related Clinical Documents can also be requested via the link https://trials.boehringer-ingelheim.com/. All requests will be governed by a Document Sharing Agreement. Bona fide, qualified scientific and medical researchers may request access to de-identified, analysable participant clinical study data with corresponding documentation describing the structure and content of the datasets. Upon approval, and governed by a Data Sharing Agreement, data are shared in a secured data-access system for a limited period of 1 year, which may be extended upon request. Researchers should use the https://trials.boehringer-ingelheim.com/ link to request access to study data.

## Abbreviations

BMI: Body mass index
CI: Confidence interval
COPD: Chronic obstructive pulmonary disorder
DPC: Diagnosis Procedure Combination
hdPS: High-dimensional propensity score
ICD-10: International Statistical Classification of Diseases and Related Health Problems, 10th Revision
ICS: Inhaled corticosteroid
IQR: Interquartile range
LABA: Long-acting β_2_-agonist
LAMA: Long-acting muscarinic antagonist
MACE: Major adverse cardiovascular event
MDV: Medical Data Vision Co., Ltd
