# Supplementary material for: Real-world effectiveness of early intervention with fixed-dose tiotropium/olodaterol vs tiotropium in Japanese patients with COPD: a high-dimensional propensity score–matched cohort analysis
Source: Respir Res. 2021 Jun 17;22:180. doi: 10.1186/s12931-021-01776-y (PMC8212527; doi:10.1186/s12931-021-01776-y)

ADDITIONAL INFORMATION

Real-world effectiveness of early intervention with fixed-dose tiotropium/olodaterol vs tiotropium in Japanese patients with COPD: a high-dimensional propensity score–matched cohort analysis

Shigeo Muro, Masaru Suzuki, Shuhei Nakamura, Jocelyn Ruoyi Wang, Elizabeth M. Garry, Wataru Sakamoto, Sabrina de Souza

[1]Department of Respiratory Medicine, Nara Medical University, Kashihara, Nara, Japan;
[2]Department of Respiratory Medicine, Faculty of Medicine, Hokkaido University, Sapporo, Japan;
[3]Nippon Boehringer Ingelheim Co., Ltd., Tokyo, Japan; [4]Science, Aetion Inc., Boston, MA, USA;
[5]Boehringer Ingelheim International GmbH, Ingelheim, Germany

Additional Table S1a Patient selection in the primary and sensitivity analysis cohorts

Additioanl Table S1b Patient selection in the sensitivity analysis cohorts for the post hoc extension study period (from 28 September 2015 to 31 March 2020)

Additional Table S2 Sensitivity analyses for escalation to triple therapy (fixed-dose or concurrent ICS/LAMA/LABA) during the prespecified study period (from 28 September 2015 to 31 March 2019)

Additional Fig. S1 Sensitivity analyses for the time-to-first COPD exacerbation in the hdPS-matched cohort during the prespecified study period (1 April 2015 to 31 March 2019). CI, confidence interval; COPD, chronic obstructive pulmonary disorder; hdPS, high-dimensional propensity score; HR, hazard ratio; SA, sensitivity analysis; Tio, tiotropium; Tio/Olo, tiotropium/olodaterol

Additional Fig. S2 Sensitivity analyses for the time-to-event for all-cause inpatient mortality, first MACE, and first use of home oxygen therapy in the hdPS-matched cohort during the prespecified study period (1 April 2015 to 31 March 2019). CI, confidence interval; hdPS, high-dimensional propensity score; HR, hazard ratio; MACE, major adverse cardiovascular event; SA, sensitivity analysis; Tio, tiotropium; Tio/Olo, tiotropium/olodaterol

Additional Table S1a Patient selection in the primary and sensitivity analysis cohorts

Reason for exclusion	Primary analysis cohort				Sensitivity analysis 1		Sensitivity analysis 2		Sensitivity analysis 3		Sensitivity analysis 4		Sensitivity analysis 5	
	Study period to 31 March 2019		Study period to 31 March 2020		Study period to 31 March 2019									
	Excluded	Remaining	Excluded	Remaining	Excluded	Remaining	Excluded	Remaining	Excluded	Remaining	Excluded	Remaining	Excluded	Remaining
All patients		29,604,920		31,898,947		29,604,920		29,604,920		29,604,920		29,604,920		29,604,920
Did not meet cohort entry criteria	-29,549,880	55,040	-31,829,413	69,534	-29,516,771	88,149	-29,549,880	55,040	-29,549,880	55,040	-29,549,887	55,033	-29,549,880	55,040
< 180 days continuous enrolment during BL period	-10,461	44,579	-13,818	55,716	-26,992	61,157	-10,461	44,579	-10,461	44,579	-10,461	44,572	-10,461	44,579
Previous use of Tio/Olo or Tio before cohort entry	-32,145	12,434	-39,822	15,894	-18,050	43,107	-32,145	12,434	-32,147	12,432	-31,699	12,873	-32,145	12,434
Age < 40 years at cohort entry	-147	12,287	-189	15,705	-873	42,234	-147	12,287	-147	12,285	-147	12,726	-147	12,287
Any LAMA/LABA/ICS maintenance therapy > 30 days during BL period	-1784	10,503	-2257	13,448	-9459	32,775	-1784	10,503	-1784	10,501	-1784	10,942	-1784	10,503
Any LAMA/LABA/ICS maintenance therapy within 30 days of cohort entry	0	10,503	0	13,448	0	32,775	0	10,503	0	10,501	0	10,942	0	10,503
No prior diagnosis COPD from the start of all available data until 1 day before cohort entry	-2985	7518	-4115	9333	-16,414	16,361	-2985	7518	-2987	7514	-2985	7957	-2985	7518
Asthma diagnosis during BL period	-367	7151	-485	8848	-1745	14,616	-367	7151	-367	7147	-81	7876	-367	7151
Lung cancer diagnosis from the start of all available data until 1 day before cohort entry	-361	6790	-468	8380	-1928	12,688	-361	6790	-361	6786	-385	7491	-361	6790
Lung transplant from the start of all available data until 1 day before cohort entry	0	6790	0	8380	0	12,688	0	6790	0	6786	0	7491	0	6790
Initiated Tio/Olo and Tio simultaneously at cohort entry	-1	6789	-7	8373	-14	12,674	-1	6789	-1	6785	-1	7490	-1	6789
Use of any triple therapy[a] during BL period or between cohort entry and 1 day before the start of the follow-up period	-1	6788	-8	8365	-5	12,669	-1	6788	-1	6784	-2	7488	-1	6788
Final cohort	-	6788		8365	-	12,669	-	6788	-	6784	-	7488	-	6788
Tio group	-	5352		6505	-	10,388	-	5352	-	5349	-	5883	-	5352
Tio/Olo group	-	1436		1860	-	2281	-	1436	-	1435	-	1605	-	1436

Sensitivity analysis 1: this cohort only included patients with one prescription for an index medication (rather than two prescriptions within 60 days)
Sensitivity analysis 2: as-treated cohort
Sensitivity analysis 3: this cohort only included patients with the COPD ICD-10 codes J43 and J44 and excluded those with the ICD-10 code J41 (chronic bronchitis)
Sensitivity analysis 4: this cohort included patients with COPD and a diagnosis code for asthma but no prescriptions for asthma-specific treatment
Sensitivity analysis 5: this cohort censored data for patients with evidence of asthma on the date of the asthma diagnosis during the follow-up period
[a]Triple therapy included any fixed-dose or concurrent use of LAMA, LABA, or ICS
BL, baseline; COPD, chronic obstructive pulmonary disorder; ICD-10, International Statistical Classification of Diseases and Related Health Problems, 10th Revision; ICS, inhaled corticosteroid; LABA, long-acting β_2-agonist; LAMA, long-acting muscarinic antagonist; Tio, tiotropium; Tio/Olo, tiotropium/olodaterol

Additional Table S1b

Patient selection in the sensitivity analysis cohorts for the post hoc extension study period (from 28 September 2015 to 31 March 2020)

Reason for exclusion	Sensitivity analysis 1		Sensitivity analysis 2		Sensitivity analysis 3		Sensitivity analysis 4		Sensitivity analysis 5	
	Study period to 31 March 2020									
	Excluded	Remaining	Excluded	Remaining	Excluded	Remaining	Excluded	Remaining	Excluded	Remaining
All patients		31,898,947		31,898,947		31,898,947		31,898,947		31,898,947
Did not meet cohort entry criteria	-31,790,245	108,702	-31,829,413	69,534	-31,829,418	69,529	-31,829,435	69,512	-31,829,413	69,534
< 180 days continuous enrolment during BL period	-25,768	82,934	-13,818	55,716	-13,818	55,711	-13,820	55,692	-13,818	55,716
Previous use of Tio/Olo or Tio before cohort entry	-47,773	35,161	-39,822	15,894	-39,825	15,886	-39,280	16,412	-39,822	15,894
Age < 40 years at cohort entry	-559	34,602	-189	15,705	-189	15,697	-189	16,223	-189	15,705
Any LAMA/LABA/ICS maintenance therapy > 30 days during BL period	-4827	29,775	-2257	13,448	-2257	13,440	-2257	13,966	-2257	13,448
Any LAMA/LABA/ICS maintenance therapy within 30 days of cohort entry	0	29,775	0	13,448	0	13,440	0	13,966	0	13,448
No prior diagnosis COPD from the start of all available data until 1 day before cohort entry	-11,866	17,909	-4115	9333	-4117	9323	-4115	9851	-4115	9333
Asthma diagnosis during BL period	-1206	16,703	-485	8848	-485	8838	-107	9744	-485	8848
Lung cancer diagnosis from the start of all available data until 1 day before cohort entry	-1161	15,542	-468	8380	-468	8370	-505	9239	-468	8380
Lung transplant from the start of all available data until 1 day before cohort entry	0	15,542	0	8380	0	8370	0	9239	0	8380
Initiated Tio/Olo and Tio simultaneously at cohort entry	-10	15,532	-7	8373	-2	8368	-2	9237	-7	8373
Use of any triple therapy[a] during BL period or between cohort entry and 1 day before the start of the follow-up period	-18	15,514	-8	8365	-8	8360	-9	9228	-8	8365
Final cohort		15,514		8365		8360		9228		8365
Tio group		12,208		6505		6502		7164		6505
Tio/Olo group		3306		1860		1858		2064		1860

Sensitivity analysis 1: this cohort only included patients with one prescription for an index medication (rather than two prescriptions within 60 days)
Sensitivity analysis 2: as-treated cohort
Sensitivity analysis 3: this cohort only included patients with the COPD ICD-10 codes J43 and J44 and excluded those with the ICD-10 code J41 (chronic bronchitis)
Sensitivity analysis 4: this cohort included patients with COPD and a diagnosis code for asthma but no prescriptions for asthma-specific treatment
Sensitivity analysis 5: this cohort censored data for patients with evidence of asthma on the date of the asthma diagnosis during the follow-up period
[a]Triple therapy included any fixed-dose or concurrent use of LAMA, LABA, or ICS
BL, baseline; COPD, chronic obstructive pulmonary disorder; ICD-10, International Statistical Classification of Diseases and Related Health Problems, 10th Revision; ICS, inhaled corticosteroid; LABA, long-acting β_2-agonist; LAMA, long-acting muscarinic antagonist; Tio, tiotropium; Tio/Olo, tiotropium/olodaterol

Additional Table S2

Sensitivity analyses for escalation to triple therapy (fixed-dose or concurrent ICS/LAMA/LABA) during the prespecified study period (from 28 September 2015 to 31 March 2019)

Variable	Sensitivity analysis 1		Sensitivity analysis 2		Sensitivity analysis 3		Sensitivity analysis 4		Sensitivity analysis 5	
	Tio	Tio/Olo	Tio	Tio/Olo	Tio	Tio/Olo	Tio	Tio/Olo	Tio	Tio/Olo
hdPS-matched cohort										
Number of patients	2183	2183	1317	1317	1304	1304	1468	1468	1314	1314
Number of escalation events	15	11	1	0	11	7	6	8	2	4
Number of patient-years	1475	1452	240	214	924	898	1036	1009	867	837
HR for time-to-escalation (95% CI)	0.74 (0.34–1.61)		NC		0.65 (0.25–1.69)		1.37 (0.47–3.95)		2.07 (0.38–11.31)	
Unmatched cohort										
Number of patients	10,388	2281	5352	1436	5349	1435	5883	1605	5352	1436
Number of escalation events	54	15	13	0	30	8	35	10	21	5
Number of patient-years	8003	1508	1178	228	4178	981	4582	1095	3846	913
HR for time-to-escalation (95% CI)	1.44 (0.81–2.54)		NC		1.11 (0.51–2.43)		1.17 (0.58–2.36)		0.98 (0.37–2.59)	

Sensitivity analysis 1: this cohort only included patients with one prescription for an index medication (rather than two prescriptions within 60 days)

Sensitivity analysis 2: as-treated cohort

Sensitivity analysis 3: this cohort only included patients with the COPD ICD-10 codes J43 and J44 and excluded those with the ICD-10 code J41 (chronic bronchitis)

Sensitivity analysis 4: this cohort included patients with COPD and a diagnosis code for asthma but no prescriptions for asthma-specific treatment

Sensitivity analysis 5: this cohort censored data for patients with evidence of asthma on the date of the asthma diagnosis during the follow-up period

CI, confidence interval; COPD, chronic obstructive pulmonary disorder; hdPS, high-dimensional propensity score; HR, hazard ratio; ICD-10, International Statistical Classification of Diseases and Related Health Problems, 10th Revision; ICS, inhaled corticosteroid; LABA, long-acting β_2-agonist; LAMA, long-acting muscarinic antagonist; NC, not calculable; Tio, tiotropium; Tio/Olo, tiotropium/olodaterol

Additional Fig. S1

Sensitivity analyses for the time-to-first COPD exacerbation in the hdPS-matched cohort during the prespecified study period (1 April 2015 to 31 March 2019). CI, confidence interval; COPD, chronic obstructive pulmonary disorder; hdPS, high-dimensional propensity score; HR, hazard ratio; SA, sensitivity analysis; Tio, tiotropium; Tio/Olo, tiotropium/olodaterol

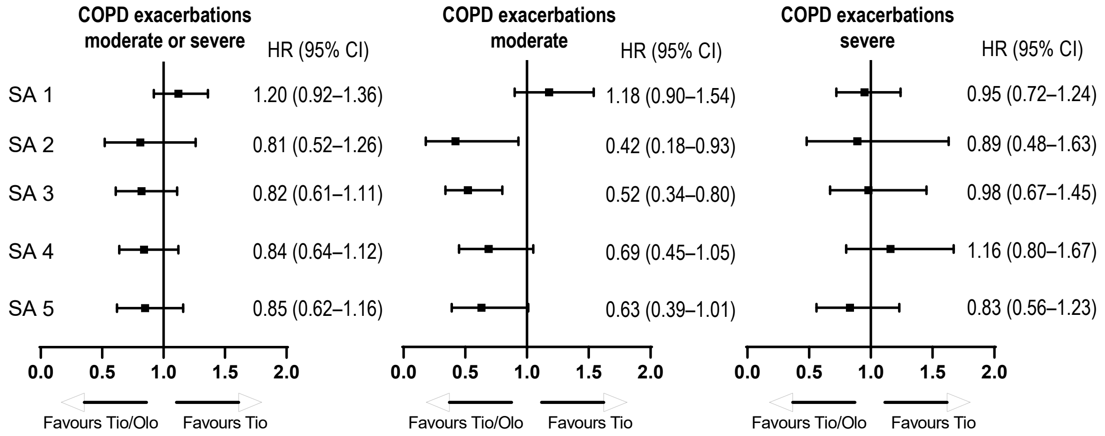

Additional Fig. S2

Sensitivity analyses for the time-to-event for all-cause inpatient mortality, first MACE, and first use of home oxygen therapy in the hdPS-matched cohort during the prespecified study period (1 April 2015 to 31 March 2019). CI, confidence interval; hdPS, high-dimensional propensity score; HR, hazard ratio; MACE, major adverse cardiovascular event; SA, sensitivity analysis; Tio, tiotropium; Tio/Olo, tiotropium/olodaterol

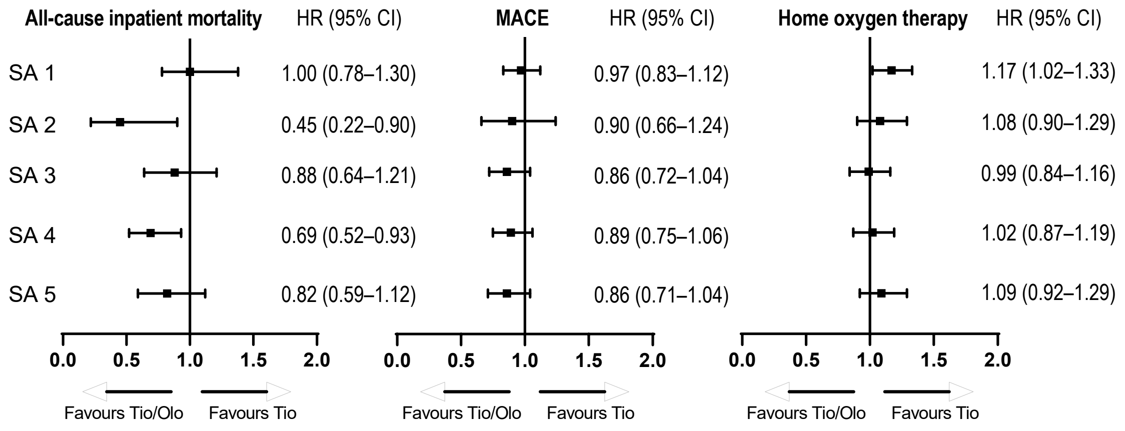

Supplement: Supplementary file 1 — Additional file 1: Table S1a. Patient selection in the primary and sensitivity analysis cohorts. Table S1b. Patient selection in the sensitivity analysis cohorts for the post hoc extension study period (from 28 September 2015 to 31 March 2020). Table S2. Sensitivity analyses for escalation to triple therapy (fixed-dose or concurrent ICS/LAMA/LABA) during the prespecified study period (from 28 September 2015 to 31 March 2019). Fig. S1. Sensitivity analyses for the time-to-first COPD exacerbation in the hdPS-matched cohort during the prespecified study period (1 April 2015 to 31 March 2019). CI, confidence interval; COPD, chronic obstructive pulmonary disorder; hdPS, high-dimensional propensity score; HR, hazard ratio; SA, sensitivity analysis; Tio, tiotropium; Tio/Olo, tiotropium/olodaterol. Fig. S2. Sensitivity analyses for the time-to-event for all-cause inpatient mortality, first MACE, and first use of home oxygen therapy in the hdPS-matched cohort during the prespecified study period (1 April 2015 to 31 March 2019). CI, confidence interval; hdPS, high-dimensional propensity score; HR, hazard ratio; MACE, major adverse cardiovascular event; SA, sensitivity analysis; Tio, tiotropium; Tio/Olo, tiotropium/olodaterol. [file 12931_2021_1776_MOESM1_ESM.pdf]
